# Sociodemographic and Health-Seeking Factors Associated with First-Trimester Prenatal Care: A Cross-Sectional Study of PRAMS Data

**DOI:** 10.3390/healthcare14020146

**Published:** 2026-01-07

**Authors:** Melissa B. Eggen, Seyed M. Karimi, Liza Creel, Bertis Little, Bridget Basile

**Affiliations:** 1 Department of Health Management and Systems Sciences, School of Public Health and Information Sciences, University of Louisville, Louisville, KY 40202, USA; melissa.eggen@louisville.edu (M.B.E.); seyed.karimi@louisville.edu (S.M.K.); bert.little@louisville.edu (B.L.); 2 Division of Health Care Policy and Research, Anschutz Medical Campus, University of Colorado, Aurora, CO 80045, USA; liza.creel@cuanschutz.edu; 3 School of Nursing, Yale University, Orange, CT 06477, USA

**Keywords:** prenatal care, pregnancy risk assessment monitoring system, Kentucky

## Abstract

**Objective**: This study assessed sociodemographic, health-seeking and social services related factors associated with first-trimester prenatal care. **Study Design**: This cross-sectional study used Phase 8 pooled data from the Kentucky Pregnancy Risk Assessment Monitoring System (PRAMS) for 2017 to 2020 and 2022. A logistic regression model was used to estimate unadjusted and adjusted odds ratios and 95% confidence intervals. **Results**: Among the 3502 women in the analytic sample, 89.3% had first-trimester prenatal care. Most respondents were White (weighted percentage, 83.3%), between the ages of 25 and 29 (31.2%), had more than a high school education (59.5%), were married (59.8%), lived in an urban area (59.4%), and had public insurance (53.9%). Maternal education was associated with the highest odds of first-trimester prenatal care, relative to other covariates, and was highest among those who completed more than high school (aOR 4.23, 95% CI 2.72–6.59) and high school (aOR 3.09, 95% CI 2.06–4.64) relative to less than high school. Private insurance, having a healthcare visit one year prior to pregnancy, and WIC receipt during pregnancy were associated with higher odds of first-trimester prenatal care. **Conclusions**: The findings of this study suggest that sociodemographic factors and access to healthcare and social services are important factors in first-trimester prenatal care.

## 1. Introduction

More than 80% of pregnancy-related deaths are deemed preventable [1]. Prenatal care offers an important opportunity to identify needed healthcare or social service supports for women and their families. First-trimester prenatal care is associated with improved perinatal outcomes, whereas late or no prenatal care receipt is associated with higher rates of adverse outcomes including maternal mortality and morbidity [2,3,4]. Clinical guidelines from the American Academy of Pediatrics (AAP) and the American College of Obstetricians and Gynecologists (ACOG) recommend that prenatal care begin in the first trimester of pregnancy and continue regularly at a frequency tailored to the needs of each pregnant woman [5]. Despite clinical recommendations, disparities in first-trimester prenatal care persist and are worsening. While most pregnant women in the United States have first-trimester prenatal care, the rate has been decreasing. From 2021 to 2023, the rate of early prenatal care decreased from 78.3% to 76.1%, while the rate of late or no prenatal care increased to 7.0% from 6.3% [6]. Contributing to these decreases are individual, structural and systemic factors such as lack of prenatal care literacy and historic and contemporary policy decisions that have weakened the maternity care workforce, resulting in variability in prenatal care timing across sociodemographic factors and geography [7,8,9]. Recognizing the decrease, Healthy People 2030 set a goal of increasing the proportion of women who receive early and adequate prenatal care to 80.5% [10].

Across the United States, rates of maternal death and severe maternal morbidity (SMM) have increased over time with marked disparities by race, socioeconomic status, and geography. Higher rates of SMM have been observed among non-Hispanic Black women with Medicaid insurance relative to non-Hispanic White women with Medicaid [11,12]. These disparities are compounded by shifts in the supply of obstetric providers, which can contribute to higher rates of late or no prenatal care, especially among those living in rural areas [13]. Maternal death has also been associated with no or late prenatal care. For example, among maternal mortalities in Kentucky in 2018, the most recent year for which prenatal care data is available, 21% had no prenatal care and 26% had one to four prenatal care visits [14]. Kentucky, a largely rural and impoverished state in the southeastern United States, has higher than average rates of maternal deaths and SMM, and is disproportionately impacted by obstetric provider shortages compared with other states [11,12,13].

To inform health policy and provide recommendations for clinical practice aimed at improving maternal and infant health outcomes, especially in states like Kentucky that are disproportionately impacted by adverse perinatal outcomes, it is essential to understand the multi-level factors that contribute to first-trimester prenatal care. We hypothesized that maternal factors associated with low socioeconomic status would be inversely related to first-trimester prenatal care, while an existing relationship with the healthcare system would be protective. The aim of this cross-sectional study was to identify maternal sociodemographic and health-seeking factors associated with first-trimester prenatal care to inform the development of strategies to facilitate access to care for those most vulnerable.

## 2. Materials & Methods

### 2.1. Study Design and Data Source

This cross-sectional analysis used pooled data from Phase 8 (2017–2020 & 2022) of the Kentucky Pregnancy Risk Assessment Monitoring System (PRAMS). PRAMS is a state-level, population-based surveillance effort conducted in partnership by the Centers for Disease Control and Prevention (CDC) and state health departments. PRAMS data includes only live births and are weighted to project population-based estimates and account for sample design, non-response, and noncoverage. PRAMS survey data are linked to the infant’s birth certificate, providing rich demographic information for both mother and infant. Additional information about PRAMS methodology is detailed elsewhere [15]. The 2021 Kentucky PRAMS data was not available due to a response rate (45.5%) that did not meet the 50% threshold required by the CDC for release [16]. Data were obtained through the Kentucky Cabinet for Health and Family Services and the University of Louisville Institutional Review Board determined this study to be exempt.

### 2.2. Sample

During the 5-year study period, 7333 women were sampled (unweighted) to participate in Kentucky PRAMS, and a total of 3940 responded for an overall response rate (unweighted) of 53.7%. Casewise deletion was used to exclude respondents who had missing values for any of the variables included in the analysis. No additional exclusion criteria were applied.

### 2.3. Measures

#### 2.3.1. Dependent Variable

The dependent variable for this study was receipt of prenatal care in the first trimester, defined as care started in the first three months of pregnancy. This variable was derived from the infant’s birth certificate where the original categories were “Yes”, “No”, and “No Prenatal Care”. Because this study focused on first-trimester prenatal care, and to avoid unstable estimates due to a small number of people with no prenatal care (n = 26), “No Prenatal Care” (n = 26) and “No” were combined to create a dichotomous outcome variable that consisted of “First-Trimester Prenatal Care” and “Late or No Prenatal Care”.

#### 2.3.2. Predictor Variables

Selection of predictor variables was informed by the current body of literature related to prenatal care timing and utilization [17,18,19].

### 2.4. Sociodemographic Characteristics

Sociodemographic variables included maternal race, age, education, marital status, rural-urban residence, previous livebirths, and pregnancy intention. Due to the relatively small number of observations of “Other” race (n = 435), compared to White and Black, maternal race was collapsed to “White” and “Non-White.” Maternal age was categorized as less than 20 years, 20–24 years, 25–29 years, 30–34 years, and 35 or older. Maternal education was categorized as less than high school, high school, or more than high school. Marital status and rural-urban residence were provided in PRAMS as dichotomous variables and were maintained as such in the analysis. The number of previous livebirths was categorized as follows: none, one, two or more. The categories of pregnancy intention were retained from the PRAMS question, “Thinking back to just before you got pregnant with your new baby, how did you feel about becoming pregnant?”.

### 2.5. Healthcare and Social Services Related Variables

Responses to the PRAMS question, “During your most recent pregnancy, what kind of health insurance did you have for your prenatal care?” were collapsed to create a binary variable for prenatal care insurance (private or public/none). Three categories (“Private health insurance from my job or the job of my husband or partner”, “Private health insurance from my parents”, and “Private health insurance from the Health Insurance Marketplace”) were collapsed into “Private”. Because there were relatively few observations with no insurance (n = 70), and because women with no insurance and public insurance often face similar barriers in access to care and social drivers of health, those with no insurance were included in the “public/none” category. Where possible, open-ended text responses in the “Other health insurance: Please tell us” were categorized into the appropriate insurance category. Enrollment in the federal Special Supplemental Nutrition Program for Women, Infants, and Children (WIC) during pregnancy was retained as a dichotomous (yes/no) variable from the birth certificate. Related to pre-pregnancy healthcare visits, PRAMS asks, “In the 12 months before you got pregnant with your new baby, did you have any health care visits with a doctor, nurse, or other health care worker, including a dental or mental health worker?”, which was retained as a dichotomous (yes/no) variable in the model.

### 2.6. Statistical Analysis

All analyses were weighted using PRAMS survey weights, which account for the complex stratified survey design, noncoverage, and nonresponse [15]. Non-weighted frequencies and weighted percentages were calculated for all predictor variables and stratified by first-trimester or late/no prenatal care. A logistic regression model was used to estimate unadjusted and adjusted odds ratios (ORs) and 95% confidence intervals associated with predictor variables. All analyses used Stata’s suite of svy commands (StataSE, College Station, TX, USA, Version 18) to project population-level estimates and account for the survey design. The subpop command in Stata was used to accurately assess variance estimates.

## 3. Results

The study sample included 3502 (unweighted) respondents, of which 3075 received first-trimester prenatal care and 427 received late or no prenatal care (Table 1). Compared to those who received late or no prenatal care, those who received first-trimester prenatal care were more likely to be White (84.7% vs. 71.4%), have more than a high school education (62.5% vs. 34.4%), be married (62.4% vs. 38.2%), have no previous live births (38.0% vs. 30.9%), wanted to be pregnant at the time they became pregnant (45.4% vs. 33.9%), have private insurance coverage (49.1% vs. 21.1%), have at least one visit with a healthcare provider in the year prior to pregnancy (72.1% vs. 46.2%), and were less likely to be enrolled in WIC during pregnancy (39.6% vs. 47.0%). Overall, 89.3% (weighted) of respondents had first-trimester prenatal care.

Table 2 shows adjusted odds ratios of first-trimester prenatal care compared to late or no prenatal care. After adjusting for sociodemographic and healthcare and social service characteristics, maternal education showed the largest adjusted odds of first-trimester prenatal care. Relative to respondents with less than a high school education, respondents with high school and more than high school had 3.09 (95% CI 2.06–4.64, *p* < 0.001) and 4.23 (95% CI 2.72–6.59, *p* < 0.001) times the odds of first-trimester prenatal care, respectively (Table 2. Prenatal care insurance and having a pre-pregnancy health visit were also strongly associated with first-trimester prenatal care. Respondents with private insurance had more than twice the odds (aOR: 2.12, 95% CI 1.34–3.37, *p* < 0.001) of having first-trimester prenatal care relative to those with public insurance. Similarly, those with a pre-pregnancy healthcare visit had twice the odds (aOR: 2.00, 95% CI 1.45–2.75, *p* < 0.001) of first-trimester prenatal care relative to those with no prior healthcare visit. Respondents who were enrolled in WIC (aOR: 1.68, 95% CI 1.18–2.39, *p* = 0.004), reported wanting to be pregnant at the time of pregnancy (aOR: 1.63, 95% CI 0.96–2.77), and were married (aOR: 1.59, 95% CI 1.09–2.34, *p* = 0.017) also had higher odds of first-trimester prenatal care relative to their counterparts.

## 4. Discussion

This study used pooled Phase 8 Kentucky PRAMS data to identify sociodemographic and health-seeking factors associated with first-trimester prenatal care. While most women in the sample had first-trimester prenatal care, nearly 11% had late or no prenatal care, which has been associated with adverse perinatal outcomes including maternal mortality and SMM [2,12,14]. The findings of this study suggest clear disparities in the receipt of first-trimester prenatal care in that those who had late or no prenatal care were more likely to be non-White, have less educational attainment, and less likely to have had a pre-pregnancy healthcare visit. The findings of this study point to factors that can be intervened upon to facilitate earlier entry to prenatal care, including engaging women in the healthcare system prior to pregnancy, and broader expansion of health policies that ensure access to care.

The Affordable Care Act (ACA) of 2010 expanded Medicaid eligibility to 138% of the Federal Poverty Level (FPL) for adults in states that adopted it. In 2014, Kentucky expanded Medicaid under the ACA and, in the same year, implemented presumptive eligibility for pregnancy, which facilitates immediate access to care for uninsured women [20]. Despite policy efforts to expand insurance coverage and increase access to healthcare for all income-eligible pregnant women, the current study found that 10.7% of women in the sample received late or no prenatal care. The findings of this study suggest that strengthening efforts to engage women in healthcare prior to pregnancy may facilitate earlier entry to prenatal care, which is important for optimal perinatal outcomes. Further, policy efforts alone may not suffice to increase rates of first-trimester prenatal care. For example, following the implementation of presumptive eligibility in Kansas, while there were small increases in earlier prenatal care, 23% of women with less than a high school education continued to not receive first-trimester prenatal care [21]. This evidence reinforces the importance of increasing community-wide awareness of presumptive eligibility for pregnancy and reducing complex administrative processes that may serve as barriers to Medicaid enrollment, particularly for women from marginalized communities [22]. Policy efforts to expand access to care, in tandem with community-based educational and advocacy efforts, can facilitate increased awareness and better outcomes.

Similar to other studies, our findings point to disparities in first-trimester prenatal care by insurance type with lower odds among those with Medicaid coverage relative to private insurance.. This finding further emphasizes the need to address social drivers of health that Medicaid-covered populations often experience in accessing prenatal care such as lack of transportation, childcare and employment barriers, and health literacy challenges [23,24]. The protective effect of enrollment in WIC, a federal safety net program designed to expand access to healthy foods and provide referrals to healthcare and other social services, highlights the role of social services in supporting entry to prenatal care, especially among women from marginalized populations. Safety net programs like WIC have the potential to strengthen women’s connections to the healthcare and social services system and provide more seamless entry to the healthcare system, which can facilitate earlier entry to prenatal care.

The results of this study should be understood in the context of its limitations. The small sample size limited the ability to obtain stable estimates for covariates that may have been influential factors for first-trimester prenatal care. For example, maternal nativity could not be included in this study due to small sample size and large amounts of missing data. The omission of relevant variables in the analysis may have produced bias in the results. PRAMS is a survey which has inherent biases, including recall and self-report bias, which may result in measurement error. Self-selection bias may have influenced the higher-than-expected rates of first-trimester prenatal care observed in our study sample compared to other data sources, which report an average (2020–2023) early prenatal care rate of 78.5% [25]. Finally, this study used Kentucky-specificdata, which may limit the generalizability of the results.

## 5. Conclusions

The findings of this study shed light on sociodemographic and health-seeking factors that can be leveraged to facilitate earlier entry to prenatal care. Health policy and community-based strategies to engage women early and often in clinical care and social services can facilitate earlier entry to prenatal care.

## Figures and Tables

**Table 1:** Sample Characteristics, Kentucky PRAMS (Phase 8), Unweighted Frequencies and Weighted Percentages *.

Characteristics	All (n = 3502)		First-Trimester PNC (n = 3075)		Late or No PNC (n = 427)		
	n	Weighted % (95% CI)	n	Weighted % (95% CI)	n	Weighted % (95% CI)	*p*-Value **
**Maternal Race**							
White	1760	83.3 (82.2–84.4)	1615	84.7 (83.6–85.8)	145	71.4 (66.0–76.2)	
Non-White	1742	16.7 (15.7–17.8)	1460	15.3 (14.2–16.4)	282	28.6 (23.8–34.0)	<0.001
**Maternal Age (Years)**							
<20	215	6.0 (5.0–7.2)	166	5.6 (4.6–6.8)	49 ^†^	9.7 (6.4–14.5)	
20–24	846	23.7 (21.8–25.6)	726	23.3 (21.4–25.3)	120	26.9 (21.4–33.2)	
25–29	1098	31.2 (29.2–33.2)	974	31.3 (29.3–33.5)	124	30.1 (24.4–36.6)	
30–34	867	25.8 (24.0–27.7)	782	26.4 (24.5–28.5)	85	20.6 (15.8–26.5)	
≥35	476	13.3 (11.9–14.8)	427	13.4 (11.9–15.0)	49 ^†^	12.6 (8.8–17.7)	0.0519
**Maternal Education**							
Less than High School	357	11.3 (10.0–12.9)	251	8.6 (7.3–10.0)	106	34.4 (28.2–41.3)	
High School	1042	29.2 (27.2–31.2)	905	29.0 (26.9–31.1)	137	31.2 (25.5–37.7)	
More than High School	2103	59.5 (57.3–61.6)	1919	62.5 (60.2–64.7)	184	34.4 (28.5–40.7)	<0.001
**Marital Status**							
Married	1702	59.8 (57.7–61.9)	1565	62.4 (60.1–64.5)	137	38.2 (31.9–44.9)	
Not Married	1800	40.2 (38.2–42.4)	1510	37.7 (35.5–39.9)	290	61.8 (55.1–68.1)	<0.001
**Maternal Residence**							
Urban	2519	59.4 (57.2–61.5)	2186	58.7 (56.4–61.0)	333	64.6 (57.8–70.8)	
Rural	982	40.6 (38.5–42.8)	889	41.0 (39.0–43.6)	94	35.4 (29.2–42.2)	0.1083
**Previous Live Births**							
None	1291	37.2 (35.1–39.3)	1149	38.0 (35.8–40.2)	142	30.9 (25.1–37.4)	
One	1112	33.1 (31.1–35.1)	1001	34.0 (31.8– 36.2)	111	25.6 (20.3–31.8)	
Two or More	1099	29.7 (27.8–31.7)	925	28.1 (26.1–30.2)	174	43.5 (37.0–50.2)	<0.001
**Pregnancy Intention**							
Wanted Later	776	18.9 (17.3–20.6)	658	18.7 (17.0–20.5)	118	20.8 (16.2–26.4)	
Wanted Sooner	414	13.7 (12.3–15.3)	379	14.1 (12.6–15.8)	35 ^†^	10.1 (6.7–15.0)	
Wanted Then	1320	44.2 (42.1–46.4)	1217	45.4 (43.2–47.7)	103	33.9 (27.8–40.7)	
Didn’t Want Then or Anytime	324	6.8 (5.8–8.0)	257	6.2 (5.2–7.3)	67	12.3 (8.6–17.3)	
Unsure What I Wanted	668	16.4 (14.9–18.1)	564	15.6 (14.1–17.4)	104	22.8 (17.8–28.9)	<0.001
**Pre-Pregnancy Health Visit**							
Yes	2368	69.4 (67.3.4–71.3)	2165	72.1 (70.0–74.2)	203	46.2 (39.7–52.9)	
No	1134	30.6 (28.7–32.7)	910	27.9 (25.8–30.0)	224	53.8 (47.1–60.3)	<0.001
**Prenatal Care Insurance**							
Public/None	2080	53.9 (51.7–56.0)	1740	50.9 (48.6–53.1)	340	78.9 (73.1–83.7)	
Private	1422	46.1 (44.0–48.3)	1335	49.1 (46.7–51.4)	87	21.1 (16.3–27.0)	<0.001
**WIC During Pregnancy**							
Yes	1551	40.4 (38.3–42.6)	1333	39.6 (37.4–41.9)	218	47.0 (40.5–53.7)	
No	1951	59.6 (57.5–61.7)	1742	60.4 (58.1–62.6)	209	53.0 (46.3–59.5)	0.0355

* Data are weighted to account for sample design, nonresponse, and noncoverage. ** *p*-value is a weighted estimate. ^†^ Estimates with 50 or fewer observations may not be generalizable.

**Table 2:** Late or No Prenatal Care (0) vs. First-Trimester Prenatal Care (1).

Covariates	Unadjusted Odds Ratios (95% CI)	*p*-Value	Adjusted Odds Ratios (95% CI)	*p*-Value
**Maternal Race (Ref: Non-White)**				
** ** White	2.22 (1.69–2.91)	<0.001	1.22 (0.88–1.69)	0.237
**Maternal Age (Years) (Ref: <20 Years)**				
** ** 20–24	1.52 (0.88–2.64)	0.136	1.17 (0.64–2.16)	0.604
** ** 25–29	1.83 (1.06–3.14)	0.029	1.06 (0.55–2.04)	0.868
** ** 30–34	2.25 (1.28–3.96)	0.005	1.10 (0.53–2.27)	0.794
** ** ≥35	1.87 (1.01–3.47)	0.048	1.17 (0.56–2.46)	0.670
**Maternal Education (Ref: Less than High School)**				
** ** High School	3.73 (2.52–5.50)	<0.001	3.09 (2.06–4.64)	<0.001
** ** More than High School	7.31 (5.06–10.6)	<0.001	4.23 (2.72–6.59)	<0.001
**Marital Status (Ref: Unmarried)**				
** ** Married	2.68 (2.00–3.59)	<0.001	1.59 (1.09–2.34)	0.017
**Maternal Residence (Ref: Urban)**				
** ** Rural	1.28 (0.95–1.73)	0.109	1.59 (1.12–2.27)	0.010
**Previous Live Births (Ref: None)**				
** ** One	1.08 (0.75–1.56)	0.684	0.95 (0.62 0 1.45)	0.812
** ** Two or More	0.53 (0.38–0.73)	<0.001	0.62 (0.40–0.96)	0.032
**Pregnancy Intention (Ref: Did not want to be pregnant then or anytime)**				
** ** Wanted to be pregnant later	1.79 (1.08–2.97)	0.023	1.50 (0.85–2.63)	0.159
** ** Wanted to be pregnant sooner	2.80 (1.52–5.14)	0.001	1.29 (0.66–2.51)	0.458
** ** Wanted to be pregnant at that time	2.68 (1.65–4.35)	<0.001	1.63 (0.96–2.77)	0.069
** ** I wasn’t sure what I wanted	1.37 (0.82–2.28)	0.225	1.10 (0.63–1.92)	0.741
**Pre-Pregnancy Health Visit (Ref: No)**				
** ** Yes	3.01 (2.26–4.01)	<0.001	2.00 (1.45–2.75)	<0.001
**Prenatal Care Insurance (Ref: Public)**				
** ** Private	3.61 (2.59–5.04)	<0.001	2.12 (1.34–3.37)	0.001
**WIC During Pregnancy (Ref: No)**				
** ** Yes	0.74 (0.56–0.98)	0.036	1.68 (1.18–2.39)	0.004

Adjusted for maternal race, age, education, marital status, maternal residence, previous live births, pregnancy intention, pre-pregnancy health visit, prenatal care insurance, and WIC during pregnancy.

## Data Availability

Restrictions apply to the availability of these data. Data were obtained from the Kentucky Department for Public Health, Division of Maternal and Child Health and can be requested through the Kentucky PRAMS Data Coordinator at the following URL: https://www.chfs.ky.gov/agencies/dph/Documents/datarg2025PRAMS.pdf#page=2.63 (accessed on 27 October 2025).
